# 4-(2-Ethyl­phen­yl)-1-(2-oxoindolin-3-yl­idene)thio­semicarbazide

**DOI:** 10.1107/S1600536810021264

**Published:** 2010-06-09

**Authors:** Humayun Pervez, Muhammad Yaqub, Muhammad Ramzan, M. Nawaz Tahir, Mohammad S. Iqbal

**Affiliations:** a Department of Chemistry, Bahauddin Zakariya University, Multan 60800, Pakistan; b Department of Physics, University of Sargodha, Sargodha, Pakistan; c Department of Chemistry, Government College University, Lahore, Pakistan

## Abstract

The title compound, C_17_H_16_N_4_OS, is stabilized in the form of a two-dimensional polymeric network due to inter­molecular N—H⋯S and N—H⋯O hydrogen bonds. An intra­molecular N—H⋯N hydrogen bond forms an *S*(5) ring, whereas inter­actions of the N—H⋯O and C—H⋯S types complete *S*(6) ring motifs. π–π inter­actions with a centroid–centroid  distance of 3.6514 (10) Å are found between the ethyl-substituted benzene ring and the heterocyclic ring of the isatin derivative.

## Related literature

For the preparation of biologically important *N* ^4^-aryl­substituted isatin-3-thio­semicarbazones, see: Pervez *et al.* (2007 ▶). For a related structure, see: Pervez *et al.* (2010 ▶): For graph-set notation, see: Bernstein *et al.* (1995 ▶).
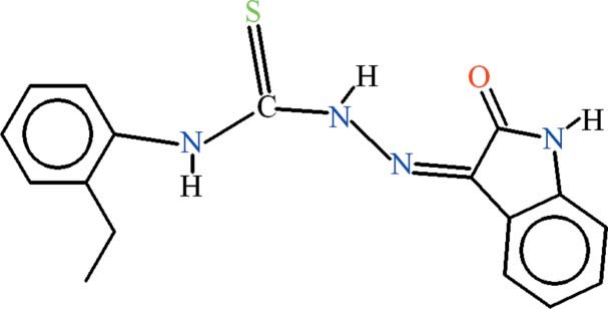


## Experimental

### 

#### Crystal data

C_17_H_16_N_4_OS
*M* *_r_* = 324.40
Monoclinic, 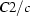

*a* = 25.6769 (7) Å
*b* = 7.4340 (2) Å
*c* = 16.6548 (5) Å
β = 96.248 (1)°
*V* = 3160.22 (15) Å^3^
*Z* = 8
Mo *K*α radiation
μ = 0.22 mm^−1^
*T* = 296 K
0.32 × 0.24 × 0.22 mm

#### Data collection

Bruker Kappa APEXII CCD diffractometer
Absorption correction: multi-scan (*SADABS*; Bruker, 2005 ▶) *T*_min_ = 0.942, *T*_max_ = 0.952
11310 measured reflections
2823 independent reflections
2400 reflections with *I* > 2σ(*I*)
*R*_int_ = 0.022

#### Refinement

*R*[*F* ^2^ > 2σ(*F* ^2^)] = 0.034
*wR*(*F* ^2^) = 0.092
*S* = 1.04
2823 reflections
209 parameters
H-atom parameters constrained
Δρ_max_ = 0.16 e Å^−3^
Δρ_min_ = −0.18 e Å^−3^

### 

Data collection: *APEX2* (Bruker, 2007 ▶); cell refinement: *SAINT* (Bruker, 2007 ▶); data reduction: *SAINT*; program(s) used to solve structure: *SHELXS97* (Sheldrick, 2008 ▶); program(s) used to refine structure: *SHELXL97* (Sheldrick, 2008 ▶); molecular graphics: *ORTEP-3 for Windows* (Farrugia, 1997 ▶) and *PLATON* (Spek, 2009 ▶); software used to prepare material for publication: *WinGX* (Farrugia, 1999 ▶) and *PLATON*.

## Supplementary Material

Crystal structure: contains datablocks global, I. DOI: 10.1107/S1600536810021264/bq2219sup1.cif

Structure factors: contains datablocks I. DOI: 10.1107/S1600536810021264/bq2219Isup2.hkl

Additional supplementary materials:  crystallographic information; 3D view; checkCIF report

## Figures and Tables

**Table 1:** Hydrogen-bond geometry (Å, °)

*D*—H⋯*A*	*D*—H	H⋯*A*	*D*⋯*A*	*D*—H⋯*A*
N1—H1⋯O1^i^	0.86	2.07	2.88 (17)	157
N3—H3*A*⋯O1	0.86	2.10	2.7711 (17)	134
N4—H4*A*⋯N2	0.86	2.13	2.5745 (18)	111
N4—H4*A*⋯S1^ii^	0.86	2.87	3.5220 (15)	133
C15—H15⋯S1	0.93	2.85	3.283 (2)	110

Symmetry codes: (i) 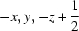; (ii) 
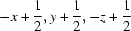
.

**Fig. 1.**
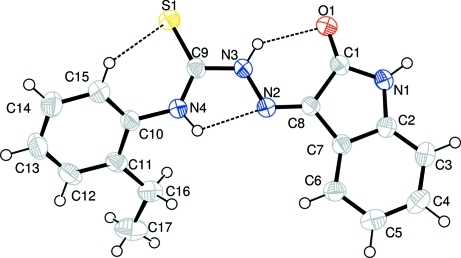
View of the title compound with the atom numbering scheme. The thermal ellipsoids are drawn at the 50% probability level. The dotted lines indicate the intra-molecular H-bondings.

**Fig. 2.**
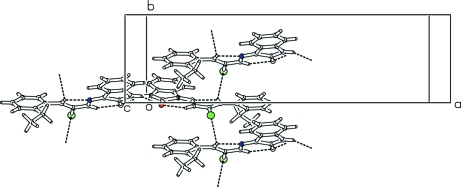
The partial packing (PLATON; Spek, 2009) which shows that molecules form two-dimensional polymeric network.

## supplementary crystallographic information

### Comment

The title compound (I), (Fig 1) is being reported in continuation of synthesizing biologically important isatin derivatives (Pervez *et al.*, 2007).

The crystal structure of (II) *i.e.* 4-(2-fluorophenyl)-1-(2-oxoindolin-3-ylidene)thiosemicarbazide (Pervez *et al.*, 2010) has been published. The title compound (I) differs from (II) due to the attachment of ethyl instead of fluoro group at position-2 of the phenyl ring substituted at N^4^ of the thiosemicarbazone moiety.

In (I) the 2-oxoindolin A (C1–C8/N1/O1), thiosemicarbazide B (N2/N3/C9/S1/N4) and phenyl ring of 2-ethylphenyl C (C10—C16) are planar with r. m. s. deviations of 0.0178, 0.0244 and 0.0149 Å, respectively. The dihedral angle between A/B, A/C and B/C is 8.71 (5)°, 33.59 (3)° and 39.32 (3)°, respectively. Due to intramolecular H-bondings (Table 1, Fig. 1), one S(5) and two S(6) (Bernstein *et al.*, 1995) ring motifs are formed. The molecules are interlinked through N—H···O and N–H···S intermolecular H-bondings. Due to these interactions infinite two-dimensional polymeric network exists. There exist π···π interaction at a distance of 3.6514 (10) Å between the benzene ring (C10—C15) and the heterocyclic ring (N1/C7/C2/C1/C8).

### Experimental

To a hot solution of isatin (0.74 g, 5.0 mmol) in ethanol (10 ml) containing a few drops of glacial acetic acid was added 4-(2-ethylphenyl)thiosemicarbazide (0.98 g, 5.0 mmol) dissolved in ethanol (10 ml) under stirring. The reaction mixture was then heated under reflux for 2 h. The yellow crystalline solid formed during heating was collected by suction filtration. Thorough washing with hot ethanol followed by ether afforded the target compound (I) in pure form (1.42 g, 88%), m. p. 485 K (*d*). The single crystals of (I) were grown in ethyl acetate by slow evaporation at room temperature.

### Refinement

The H-atoms were positioned geometrically (N–H = 0.86 Å, C–H = 0.93–0.97 Å) and refined as riding with *U*_iso_(H) = *xU*_eq_(C, N), where *x* = 1.5 for methyl and *x* = 1.2 for all other H-atoms.

### Crystal data

**Table:**

C_17_H_16_N_4_OS	*F*(000) = 1360
*M**_r_* = 324.40	*D*_x_ = 1.364 Mg m^−^^3^
Monoclinic, *C*2/*c*	Mo *K*α radiation, λ = 0.71073 Å
Hall symbol: -C 2yc	Cell parameters from 2400 reflections
*a* = 25.6769 (7) Å	θ = 2.9–25.3°
*b* = 7.4340 (2) Å	µ = 0.22 mm^−^^1^
*c* = 16.6548 (5) Å	*T* = 296 K
β = 96.248 (1)°	Prism, yellow
*V* = 3160.22 (15) Å^3^	0.32 × 0.24 × 0.22 mm
*Z* = 8	

### Data collection

**Table:**

Bruker Kappa APEXII CCD diffractometer	2823 independent reflections
Radiation source: fine-focus sealed tube	2400 reflections with *I* > 2σ(*I*)
graphite	*R*_int_ = 0.022
Detector resolution: 8.10 pixels mm^-1^	θ_max_ = 25.3°, θ_min_ = 2.9°
ω scans	*h* = −30→30
Absorption correction: multi-scan (*SADABS*; Bruker, 2005)	*k* = −7→8
*T*_min_ = 0.942, *T*_max_ = 0.952	*l* = −19→19
11310 measured reflections	

### Refinement

**Table:**

Refinement on *F*^2^	Primary atom site location: structure-invariant direct methods
Least-squares matrix: full	Secondary atom site location: difference Fourier map
*R*[*F*^2^ > 2σ(*F*^2^)] = 0.034	Hydrogen site location: inferred from neighbouring sites
*wR*(*F*^2^) = 0.092	H-atom parameters constrained
*S* = 1.04	*w* = 1/[σ^2^(*F*_o_^2^) + (0.0406*P*)^2^ + 2.640*P*] where *P* = (*F*_o_^2^ + 2*F*_c_^2^)/3
2823 reflections	(Δ/σ)_max_ < 0.001
209 parameters	Δρ_max_ = 0.16 e Å^−^^3^
0 restraints	Δρ_min_ = −0.18 e Å^−^^3^

### Special details

**Table:**

Geometry. Bond distances, angles *etc*. have been calculated using the rounded fractional coordinates. All su's are estimated from the variances of the (full) variance-covariance matrix. The cell e.s.d.'s are taken into account in the estimation of distances, angles and torsion angles
Refinement. Refinement of *F*^2^ against ALL reflections. The weighted *R*-factor *wR* and goodness of fit *S* are based on *F*^2^, conventional *R*-factors *R* are based on *F*, with *F* set to zero for negative *F*^2^. The threshold expression of *F*^2^ > σ(*F*^2^) is used only for calculating *R*-factors(gt) *etc*. and is not relevant to the choice of reflections for refinement. *R*-factors based on *F*^2^ are statistically about twice as large as those based on *F*, and *R*- factors based on ALL data will be even larger.

### Fractional atomic coordinates and isotropic or equivalent isotropic displacement parameters (Å^2^)

**Table:**

	*x*	*y*	*z*	*U*_iso_*/*U*_eq_	
S1	0.23890 (2)	−0.14366 (7)	0.38931 (2)	0.0442 (2)	
O1	0.06872 (4)	−0.02675 (19)	0.27866 (7)	0.0479 (4)	
N1	0.02670 (5)	0.0792 (2)	0.15804 (8)	0.0442 (5)	
N2	0.16415 (5)	0.02887 (19)	0.19012 (8)	0.0349 (4)	
N3	0.17539 (5)	−0.03419 (19)	0.26578 (8)	0.0372 (4)	
N4	0.26102 (5)	−0.0071 (2)	0.24633 (8)	0.0393 (4)	
C1	0.06870 (6)	0.0291 (2)	0.20906 (10)	0.0381 (5)	
C2	0.04184 (6)	0.1426 (2)	0.08419 (10)	0.0391 (5)	
C3	0.01121 (7)	0.2144 (3)	0.01920 (11)	0.0516 (6)	
C4	0.03640 (8)	0.2668 (3)	−0.04645 (11)	0.0547 (7)	
C5	0.09005 (8)	0.2485 (3)	−0.04709 (11)	0.0495 (6)	
C6	0.12070 (7)	0.1798 (2)	0.01934 (9)	0.0409 (5)	
C7	0.09608 (6)	0.1274 (2)	0.08544 (9)	0.0341 (5)	
C8	0.11566 (6)	0.0567 (2)	0.16408 (9)	0.0333 (5)	
C9	0.22671 (6)	−0.0580 (2)	0.29697 (9)	0.0342 (5)	
C10	0.31682 (6)	−0.0090 (2)	0.25921 (10)	0.0365 (5)	
C11	0.34405 (6)	−0.0684 (2)	0.19598 (10)	0.0392 (5)	
C12	0.39859 (7)	−0.0623 (3)	0.20944 (12)	0.0511 (7)	
C13	0.42445 (7)	0.0001 (3)	0.28101 (13)	0.0585 (7)	
C14	0.39670 (7)	0.0588 (3)	0.34154 (12)	0.0546 (7)	
C15	0.34255 (7)	0.0550 (3)	0.33083 (11)	0.0452 (6)	
C16	0.31486 (8)	−0.1327 (3)	0.11775 (11)	0.0495 (6)	
C17	0.34737 (10)	−0.2230 (3)	0.05832 (13)	0.0717 (9)	
H1	−0.00519	0.07323	0.16915	0.0531*	
H3	−0.02481	0.22716	0.01929	0.0619*	
H3A	0.15051	−0.05950	0.29454	0.0446*	
H4	0.01673	0.31566	−0.09139	0.0656*	
H4A	0.24765	0.03177	0.19999	0.0471*	
H5	0.10561	0.28267	−0.09261	0.0594*	
H6	0.15681	0.16924	0.01957	0.0490*	
H12	0.41813	−0.10153	0.16896	0.0613*	
H13	0.46088	0.00228	0.28809	0.0702*	
H14	0.41418	0.10101	0.38969	0.0655*	
H15	0.32347	0.09524	0.37168	0.0543*	
H16A	0.29714	−0.03025	0.09104	0.0595*	
H16B	0.28807	−0.21671	0.13056	0.0595*	
H17A	0.37156	−0.13760	0.04015	0.1076*	
H17B	0.32467	−0.26665	0.01289	0.1076*	
H17C	0.36643	−0.32188	0.08436	0.1076*	

### Atomic displacement parameters (Å^2^)

**Table:**

	*U*^11^	*U*^22^	*U*^33^	*U*^12^	*U*^13^	*U*^23^
S1	0.0421 (3)	0.0538 (3)	0.0371 (2)	0.0059 (2)	0.0058 (2)	0.0088 (2)
O1	0.0369 (6)	0.0701 (9)	0.0379 (7)	0.0024 (6)	0.0097 (5)	0.0099 (6)
N1	0.0265 (7)	0.0667 (10)	0.0396 (8)	−0.0015 (7)	0.0045 (6)	0.0036 (7)
N2	0.0306 (7)	0.0406 (8)	0.0334 (7)	0.0011 (6)	0.0036 (5)	0.0002 (6)
N3	0.0290 (7)	0.0488 (9)	0.0345 (7)	0.0018 (6)	0.0061 (5)	0.0052 (6)
N4	0.0294 (7)	0.0548 (9)	0.0339 (7)	0.0038 (6)	0.0043 (5)	0.0074 (6)
C1	0.0321 (8)	0.0449 (10)	0.0379 (9)	−0.0012 (7)	0.0060 (7)	−0.0007 (7)
C2	0.0345 (8)	0.0455 (10)	0.0368 (8)	−0.0021 (7)	0.0013 (7)	−0.0010 (7)
C3	0.0384 (9)	0.0649 (13)	0.0495 (10)	0.0047 (9)	−0.0042 (8)	0.0045 (9)
C4	0.0584 (12)	0.0610 (13)	0.0418 (10)	0.0041 (10)	−0.0071 (9)	0.0092 (9)
C5	0.0600 (12)	0.0511 (12)	0.0378 (9)	−0.0038 (9)	0.0069 (8)	0.0048 (8)
C6	0.0425 (9)	0.0449 (10)	0.0356 (9)	−0.0032 (8)	0.0063 (7)	−0.0013 (7)
C7	0.0331 (8)	0.0358 (9)	0.0329 (8)	−0.0007 (7)	0.0020 (6)	−0.0035 (7)
C8	0.0304 (8)	0.0374 (9)	0.0325 (8)	−0.0002 (7)	0.0049 (6)	−0.0018 (7)
C9	0.0314 (8)	0.0344 (9)	0.0372 (9)	0.0042 (6)	0.0053 (6)	−0.0032 (7)
C10	0.0317 (8)	0.0386 (9)	0.0394 (9)	0.0028 (7)	0.0054 (7)	0.0084 (7)
C11	0.0408 (9)	0.0359 (9)	0.0424 (9)	0.0052 (7)	0.0118 (7)	0.0102 (7)
C12	0.0419 (10)	0.0567 (12)	0.0583 (12)	0.0099 (9)	0.0214 (9)	0.0154 (9)
C13	0.0308 (9)	0.0763 (15)	0.0682 (13)	−0.0005 (9)	0.0051 (9)	0.0178 (11)
C14	0.0410 (10)	0.0684 (14)	0.0523 (11)	−0.0099 (9)	−0.0040 (8)	0.0095 (10)
C15	0.0391 (9)	0.0541 (11)	0.0426 (10)	−0.0009 (8)	0.0048 (7)	0.0029 (8)
C16	0.0587 (11)	0.0465 (11)	0.0448 (10)	0.0051 (9)	0.0119 (8)	0.0032 (8)
C17	0.0976 (17)	0.0655 (15)	0.0572 (13)	−0.0001 (13)	0.0317 (12)	−0.0070 (11)

### Geometric parameters (Å, °)

**Table:**

S1—C9	1.6625 (15)	C10—C15	1.384 (2)
O1—C1	1.231 (2)	C11—C16	1.508 (3)
N1—C1	1.351 (2)	C11—C12	1.395 (2)
N1—C2	1.411 (2)	C12—C13	1.380 (3)
N2—N3	1.3460 (19)	C13—C14	1.368 (3)
N2—C8	1.290 (2)	C14—C15	1.383 (3)
N3—C9	1.374 (2)	C16—C17	1.519 (3)
N4—C9	1.339 (2)	C3—H3	0.9300
N4—C10	1.426 (2)	C4—H4	0.9300
N1—H1	0.8600	C5—H5	0.9300
N3—H3A	0.8600	C6—H6	0.9300
N4—H4A	0.8600	C12—H12	0.9300
C1—C8	1.501 (2)	C13—H13	0.9300
C2—C7	1.395 (2)	C14—H14	0.9300
C2—C3	1.375 (2)	C15—H15	0.9300
C3—C4	1.386 (3)	C16—H16A	0.9700
C4—C5	1.386 (3)	C16—H16B	0.9700
C5—C6	1.384 (2)	C17—H17A	0.9600
C6—C7	1.384 (2)	C17—H17B	0.9600
C7—C8	1.450 (2)	C17—H17C	0.9600
C10—C11	1.398 (2)		
			
S1···C15	3.283 (2)	C1···H1^iii^	2.7600
S1···N4^i^	3.5220 (15)	C1···H3A	2.4900
S1···H15	2.8500	C5···H17A^vii^	3.0500
S1···H6^ii^	3.1900	C9···H15	2.8900
S1···H4A^i^	2.8700	C9···H16B^iv^	2.8500
S1···H16A^i^	3.0500	C12···H5^vii^	2.8400
O1···N2	3.0226 (17)	C12···H17A	2.8800
O1···N3	2.7711 (17)	C12···H17C	2.8900
O1···C5^ii^	3.331 (2)	C16···H4A	2.6200
O1···N1^iii^	2.8805 (17)	C17···H12	2.6000
O1···H1^iii^	2.0700	H1···O1^iii^	2.0700
O1···H3A	2.1000	H1···C1^iii^	2.7600
N1···O1^iii^	2.8805 (17)	H3A···O1	2.1000
N2···O1	3.0226 (17)	H3A···C1	2.4900
N2···N4	2.5745 (18)	H4A···N2	2.1300
N3···O1	2.7711 (17)	H4A···C16	2.6200
N4···C9^iv^	3.437 (2)	H4A···H16A	2.3700
N4···N2	2.5745 (18)	H4A···H16B	2.4700
N4···S1^iv^	3.5220 (15)	H4A···S1^iv^	2.8700
N2···H4A	2.1300	H5···C12^vii^	2.8400
N4···H16B	2.6300	H6···S1^vi^	3.1900
N4···H16A	2.8500	H12···C17	2.6000
C1···C13^iv^	3.509 (3)	H12···H17A	2.3500
C1···C12^iv^	3.395 (3)	H12···H17C	2.4500
C2···C3^v^	3.370 (3)	H13···H13^viii^	2.4900
C2···C13^iv^	3.526 (3)	H15···S1	2.8500
C3···C3^v^	3.290 (3)	H15···C9	2.8900
C3···C2^v^	3.370 (3)	H16A···N4	2.8500
C5···O1^vi^	3.331 (2)	H16A···H4A	2.3700
C7···C14^iv^	3.428 (3)	H16A···S1^iv^	3.0500
C8···C12^iv^	3.572 (3)	H16B···N4	2.6300
C9···N4^i^	3.437 (2)	H16B···H4A	2.4700
C12···C8^i^	3.572 (3)	H16B···C9^i^	2.8500
C12···C1^i^	3.395 (3)	H17A···C12	2.8800
C13···C2^i^	3.526 (3)	H17A···H12	2.3500
C13···C1^i^	3.509 (3)	H17A···C5^vii^	3.0500
C14···C7^i^	3.428 (3)	H17C···C12	2.8900
C15···S1	3.283 (2)	H17C···H12	2.4500
			
C1—N1—C2	111.33 (13)	C12—C11—C16	123.02 (16)
N3—N2—C8	118.13 (13)	C11—C12—C13	122.01 (17)
N2—N3—C9	119.86 (13)	C12—C13—C14	120.24 (17)
C9—N4—C10	128.46 (14)	C13—C14—C15	119.71 (18)
C2—N1—H1	124.00	C10—C15—C14	119.83 (17)
C1—N1—H1	124.00	C11—C16—C17	116.51 (17)
C9—N3—H3A	120.00	C2—C3—H3	121.00
N2—N3—H3A	120.00	C4—C3—H3	121.00
C9—N4—H4A	116.00	C3—C4—H4	119.00
C10—N4—H4A	116.00	C5—C4—H4	119.00
O1—C1—N1	127.14 (14)	C4—C5—H5	120.00
O1—C1—C8	126.71 (14)	C6—C5—H5	120.00
N1—C1—C8	106.14 (13)	C5—C6—H6	121.00
N1—C2—C7	109.23 (13)	C7—C6—H6	121.00
C3—C2—C7	121.86 (15)	C11—C12—H12	119.00
N1—C2—C3	128.89 (15)	C13—C12—H12	119.00
C2—C3—C4	117.02 (17)	C12—C13—H13	120.00
C3—C4—C5	121.90 (18)	C14—C13—H13	120.00
C4—C5—C6	120.63 (17)	C13—C14—H14	120.00
C5—C6—C7	118.08 (17)	C15—C14—H14	120.00
C2—C7—C6	120.47 (15)	C10—C15—H15	120.00
C2—C7—C8	106.88 (13)	C14—C15—H15	120.00
C6—C7—C8	132.64 (15)	C11—C16—H16A	108.00
N2—C8—C7	126.10 (14)	C11—C16—H16B	108.00
N2—C8—C1	127.49 (14)	C17—C16—H16A	108.00
C1—C8—C7	106.38 (13)	C17—C16—H16B	108.00
S1—C9—N3	118.38 (12)	H16A—C16—H16B	107.00
S1—C9—N4	128.35 (12)	C16—C17—H17A	109.00
N3—C9—N4	113.27 (13)	C16—C17—H17B	109.00
C11—C10—C15	121.82 (15)	C16—C17—H17C	109.00
N4—C10—C15	120.28 (15)	H17A—C17—H17B	109.00
N4—C10—C11	117.83 (14)	H17A—C17—H17C	109.00
C10—C11—C12	116.40 (15)	H17B—C17—H17C	109.00
C10—C11—C16	120.58 (15)		
			
C1—N1—C2—C3	−176.03 (18)	C2—C3—C4—C5	−0.1 (3)
C2—N1—C1—O1	177.74 (16)	C3—C4—C5—C6	−1.3 (3)
C2—N1—C1—C8	−1.78 (17)	C4—C5—C6—C7	1.1 (3)
C1—N1—C2—C7	2.28 (18)	C5—C6—C7—C8	−177.87 (17)
N3—N2—C8—C7	178.61 (14)	C5—C6—C7—C2	0.5 (2)
C8—N2—N3—C9	−177.12 (14)	C2—C7—C8—N2	−177.51 (15)
N3—N2—C8—C1	0.9 (2)	C6—C7—C8—N2	1.1 (3)
N2—N3—C9—S1	−177.65 (12)	C6—C7—C8—C1	179.21 (16)
N2—N3—C9—N4	2.2 (2)	C2—C7—C8—C1	0.65 (16)
C10—N4—C9—S1	−1.7 (3)	N4—C10—C11—C12	177.69 (16)
C9—N4—C10—C15	−45.5 (2)	N4—C10—C11—C16	−1.6 (2)
C10—N4—C9—N3	178.48 (15)	C15—C10—C11—C12	1.0 (2)
C9—N4—C10—C11	137.74 (17)	C15—C10—C11—C16	−178.32 (17)
O1—C1—C8—N2	−0.7 (3)	N4—C10—C15—C14	−177.59 (18)
O1—C1—C8—C7	−178.83 (16)	C11—C10—C15—C14	−0.9 (3)
N1—C1—C8—N2	178.80 (16)	C10—C11—C12—C13	−0.4 (3)
N1—C1—C8—C7	0.68 (16)	C16—C11—C12—C13	178.81 (19)
N1—C2—C3—C4	179.90 (17)	C10—C11—C16—C17	−170.19 (16)
C7—C2—C3—C4	1.8 (3)	C12—C11—C16—C17	10.6 (3)
N1—C2—C7—C6	179.51 (14)	C11—C12—C13—C14	−0.1 (3)
N1—C2—C7—C8	−1.72 (17)	C12—C13—C14—C15	0.2 (3)
C3—C2—C7—C6	−2.0 (2)	C13—C14—C15—C10	0.4 (3)
C3—C2—C7—C8	176.74 (16)		

Symmetry codes: (i) −*x*+1/2, *y*−1/2, −*z*+1/2; (ii) *x*, −*y*, *z*+1/2; (iii) −*x*, *y*, −*z*+1/2; (iv) −*x*+1/2, *y*+1/2, −*z*+1/2; (v) −*x*, −*y*, −*z*; (vi) *x*, −*y*, *z*−1/2; (vii) −*x*+1/2, −*y*+1/2, −*z*; (viii) −*x*+1, *y*, −*z*+1/2.

### Hydrogen-bond geometry (Å, °)

**Table:**

*D*—H···*A*	*D*—H	H···*A*	*D*···*A*	*D*—H···*A*
N1—H1···O1^iii^	0.8600	2.0700	2.8805 (17)	157.00
N3—H3A···O1	0.8600	2.1000	2.7711 (17)	134.00
N4—H4A···N2	0.8600	2.1300	2.5745 (18)	111.00
N4—H4A···S1^iv^	0.8600	2.8700	3.5220 (15)	133.00
C15—H15···S1	0.9300	2.8500	3.283 (2)	110.00

Symmetry codes: (iii) −*x*, *y*, −*z*+1/2; (iv) −*x*+1/2, *y*+1/2, −*z*+1/2.
